# Studying the urine microbiome in superficial bladder cancer: samples obtained by midstream voiding versus cystoscopy

**DOI:** 10.1186/s12894-020-0576-z

**Published:** 2020-01-28

**Authors:** Suchitra K. Hourigan, Wei Zhu, Wendy S.W.Wong, Nicole C. Clemency, Marina Provenzano, Thierry Vilboux, John E. Niederhuber, John Deeken, Simon Chung, Kim McDaniel-Wiley, Donald Trump

**Affiliations:** 1 Inova Children’s Hospital, 3300 Gallows Road, Falls Church, VA 22042 USA; 2 Inova Translational Medicine Institute, 3300 Gallows Road, Falls Church, VA 22042 USA; 3 Public Health Sciences, Center for Genomics in Public Health, School of Medicine, University of Virginia, Charlottesville, VA 22908 USA; 4 Inova Schar Cancer Institute, 3224 Gallows Road, Fairfax, VA 22031 USA; 5 Department of Urology, Inova Fairfax Medical Center, 3300 Gallows Road, Falls Church, VA 22042 USA

**Keywords:** Urine, Microbiome, Cystoscopy

## Abstract

**Background:**

Preliminary data suggest that the urinary microbiome may play a role in bladder cancer. Information regarding the most suitable method of collecting urine specimens is needed for the large population studies needed to address this. To compare microbiome metrics resulting from 16S ribosomal RNA gene sequencing between midstream, voided specimens and those obtained at cystoscopy.

**Methods:**

Adults, with a history of superficial urothelial cell carcinoma (non-muscle invasive bladder cancer) being followed with periodic surveillance cystoscopy had a urine sample collected by a mid-stream, voided technique and then from the bladder at cystoscopy. Urine samples underwent 16S ribosomal RNA gene sequencing on the Illumina MiSeq platform.

**Results:**

22 subjects (8 female, 14 male) were included. There was no significant difference in beta diversity (diversity between samples) in all samples between collection methods. However, analysis by sex revealed a difference between voided and cystoscopy samples from the same individual in males (*p* = 0.006, Adonis test) but not in females (*p* = 0.317, Adonis test). No differences were seen by collection method in any alpha diversity (diversity within a sample) measurement or differential abundance of taxa.

**Conclusions:**

Beta diversity of the urine microbiome did differ by collection method for males only. This suggests that the urinary microbiomes of the two collection methods are not equivalent to each other, at least in males, which is the sex that bladder cancer occurs most frequently in. Therefore, the same collection method within a given study should be used.

## Background

It is increasingly clear that the urinary tract, once considered in healthy individuals to be a sterile body niche, on more specialized analysis maintains a microbial population. The role of these populations and the impact of changes in them in various genitourinary conditions is still being defined [1]. Changes in the urinary tract microbiome have been observed among individuals with urinary incontinence,, neurogenic bladder dysfunction, sexually transmitted infections, benign prostatic hyperplasia and lower urinary tract symptoms [2–7].

Urothelial carcinoma (UC) is a major health problem, accounting for approximately 75,000 new cases annually and resulting in 16,000 cancer deaths each year in the Unites States [8]. Muscle invading bladder cancer, which accounts for approximately 30% of new cases of UC each year is by far the most dangerous form of UC, with a 50% mortality rate at 5 years. Superficial UC, which is usually not life threatening, has a substantial risk to recur after initial treatment and hence requires regular surveillance cystoscopy and often retreatment markedly increasing the inconvenience, risk and cost. It is plausible to hypothesize that changes or differences in the urinary microbiome could be associated with different risk of superficial or muscle-invading cancer as well as the risk of recurrence of superficial disease. Many studies have examined the relationship between the host microbiome and cancer susceptibility in systems other than the urinary tract [9]. Many studies have focused on the association of intestinal microbiota and cancers of the gastrointestinal tract. There are, however, few studies of the urinary microbiome in individuals with UC and the role of the urinary microbiome in bladder cancer has not yet been elucidated. Nevertheless, there are intriguing potential relationships between urinary tract microorganisms and UC: (i) schistosomal infection is clearly associated with an increased risk of UC, intriguingly most often of squamous histology, a relatively uncommon histology among all individuals with UC [10] (ii) carcinogen exposure of the urinary epithelium is clearly a cause of UC in experimental and human studies; it is plausible that there may be a role of bacterial communities in the urinary tract influencing the metabolism of carcinogenic compounds and increasing or decreasing the risk of UC [11, 12] and (iii) the role of Bacillus Calmette-Guerin, an attenuated *Mycobacterium tuberculosis* in the treatment of superficial UC may not be just from inducing inflammation [13]; local microbiota may directly inactivate BCG in the bladder or modulate urothelial sensitivity to BCG [14]. Overall the bladder microbiome may also promote or inhibit UC pathogenesis and progression [14]. Xu and colleagues in a small study of patients with UC (*n* = 8) found differences in the urine microbiome compared to healthy individuals, such as enrichment of the genus *Streptococcus* in patients’ urine [1]. Bao et al. reported differences in the urine microbiome among patients in whom superficial UC recurred compared to those in whom it did not [15].

Voided, mid-stream urine samples are traditionally thought to be representative of the bladder in both males and females minimizing distal urethral contamination and are relatively easy to collect for large epidemiological studies [16]. However, it has been shown voided samples contained genital tract bacteria in females, and more recently that catheterized samples contained fewer lower urinary tract microbes than voided samples in males [5, 17] However, in the bladder cancer population, it is unknown what the microbiome differences are in voided urine samples versus urine samples obtained directly from the bladder (i.e. obtained by urinary catheterization or at cystoscopy). Delineating the differences, if any, between these two methods of urine sampling will be important in designing studies of large numbers of individuals which will be necessary to elucidate the role of the urine microbiome in bladder cancer.

### Aim

To compare microbiome metrics resulting from 16S ribosomal (r) RNA gene sequencing between urine obtained from mid-stream urine (voided) and cystoscopy among a population of individuals undergoing regular surveillance cystoscopy because of a prior history of superficial UC.

## Methods

Informed written consent to obtain mid-stream voided urine specimens and immediately thereafter a urine specimen from the bladder at the time of cystoscopy was obtained individuals undergoing surveillance cystoscopy for a prior history of superficial UC defined as non-muscle invasive bladder cancer (IRB# 15–1986). Inclusion criteria were 1) History of non-muscle invasive bladder cancer undergoing routine surveillance cystoscopy; 2) > 180 days since last intra-vesical therapy; 3) > 180 days since last urethral instrumentation; 4) No current symptoms or signs of an active urinary tract infection; 5) > 18 years of age; 6) No history of urinary tract stones and 7) No nephrostomy or bladder catheter in place. Exclusion criteria were 1) Failure to satisfy inclusion criteria and 2) Inability to provide informed consent.

Standard technique for mid-stream collection was employed for men and women. Cystoscopy was then performed. Intraurethral lubricant jelly was used for all subjects for comfort and safety. No subject received periprocedural antibiotics. Immediately after insertion of the cystoscope a 20 cc aliquot of urine was removed. Urine samples were stored within 90 min of collection at − 80 °C until analysis. Clinical data was collected including demographics and recent antibiotic use.

Prior to DNA extraction, 1–1.5 ml of urine was thawed and centrifuged at 20,000 x g for 3 min. The supernatants were discarded and the pellets were resuspended in 400 μL of Buffer ATL (Qiagen, CA) and treated with 25 μL of lysozyme solution (50 mg/mL) (Sigma Aldridge, MO). Samples were heated for 5 min at 70 °C and cooled before loading on the EZ1 Advanced (Qiagen) for DNA extraction by using the EZ1 DSP Virus kit (Qiagen). Samples were then cleaned and concentrated using the DNeasy PowerClean Cleanup Kit (Qiagen). Sequencing was prepared using a Nextera XT kit according to the Illumina 16S Metagenomic Sequencing Library Preparation protocol for analysis of hypervariable regions V3-V4. The locus specific sequences (Illumina, CA) using standard IUPAC nucleotide nomenclature were: 16S Amplicon PCR Forward Primer = 5′- GACTACHVGGGTATCTAATCC -3′, 16S Amplicon PCR Reverse Primer = 5′- CCTACGGGNGGCWGCAG − 3′.For normalization, each library was quantified with the KAPA Library Quantification Kit (Kapa Biosystems, MA). The libraries were sequenced on the Illumina MiSeq with paired-end 300 bp reads.

Three control samples were included; *Staphylococcus aureus* (Strain NCTC 8532, ATCC, VA)*, Escherichia coli* (Strain NCTC 9001, ATCC, VA) and a “blank” control of 400 μL of buffer ATL which underwent the same DNA extraction process as above. Quantification of the library of the negative control was below the recommended concentration of 4 nM.

Qiime 1.9 was used to demultiplex fastq files, for open-reference Operational Taxonomic Unit (OTU) picking and building the phylogenetic tree using the open reference method [18]. The OTU table and the tree were then imported into R packages phyloseq [19] (version 1.19.1) and vegan (version 2.4–3) for analysis. Similar to our previous microbiome study, OTUs that were not seen more than three times in at least 20% of the samples were removed [20]. Samples with low read counts (< 5,000) and subjects with unpaired samples (i.e. not having both a voided and a cystoscopy sample) were removed. Rarefaction was performed on the remaining samples to be able to compare species richness.

Alpha diversity measures (i.e., observed species, Shannon, Simpson, Fisher) and beta diversity measures (unweighted UniFrac Distance, weighted UniFrac Distance, Bray-Curtis, and Jensen-Shannon divergence) were evaluated between voided samples and those collected by cystoscopy.

The 16S rRNA sequence data is deposited in NCBI Sequence Read Archive (SRA), PRJNA600265.

## Results

After sample filtering was applied (see methods), 8 samples were removed for low read counts (< 5000) and 5 for unmatched samples, leaving 22 subjects with paired samples (both voided and collected by cystoscopy) that were included. Clinical and demographic features of the study population are shown in Table 1. Females were younger than males (mean age female 68.5 years versus mean age male 76.8 years, *p* = 0.004). 4 subjects had antibiotic use within the three months prior to urine sample collection; these were all female.

**Table 1:** Clinical and demographics features of subjects

	Female	Male	P
Number	8	14	
Mean age in years (SD)	68.5 (6.7)	76.8 (5.2)	**0.004**
Smoking history – yes (%)	5 (50)	8 (57.1)	1
Mean body mass index, kg/m^2^ (SD)	27.1 (5.9)	28.2 (3.3)	0.602
Antibiotic use in last 3 months –yes (%)	4 (40)	0 (0)	**0.024**

**Fig. 1:**
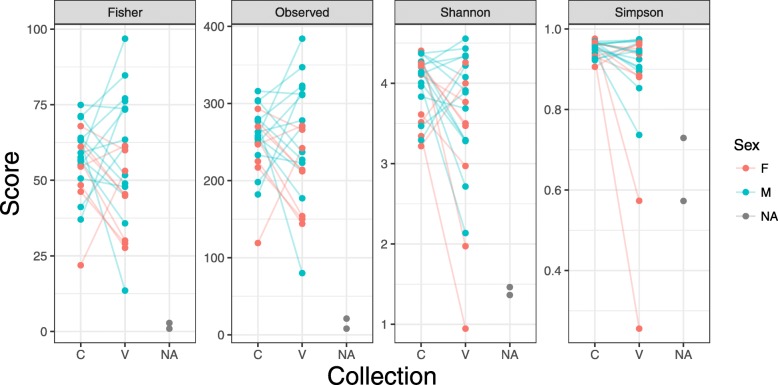
Alpha diversity of voided (V) versus cystoscopy (C) urine samples; lines connect samples from the same individual. NA represents control samples

Among all subjects there was no significant difference in any alpha diversity measure between collection methods (voided versus cystoscopy) or sex (Fig. 1).

**Fig. 2:**
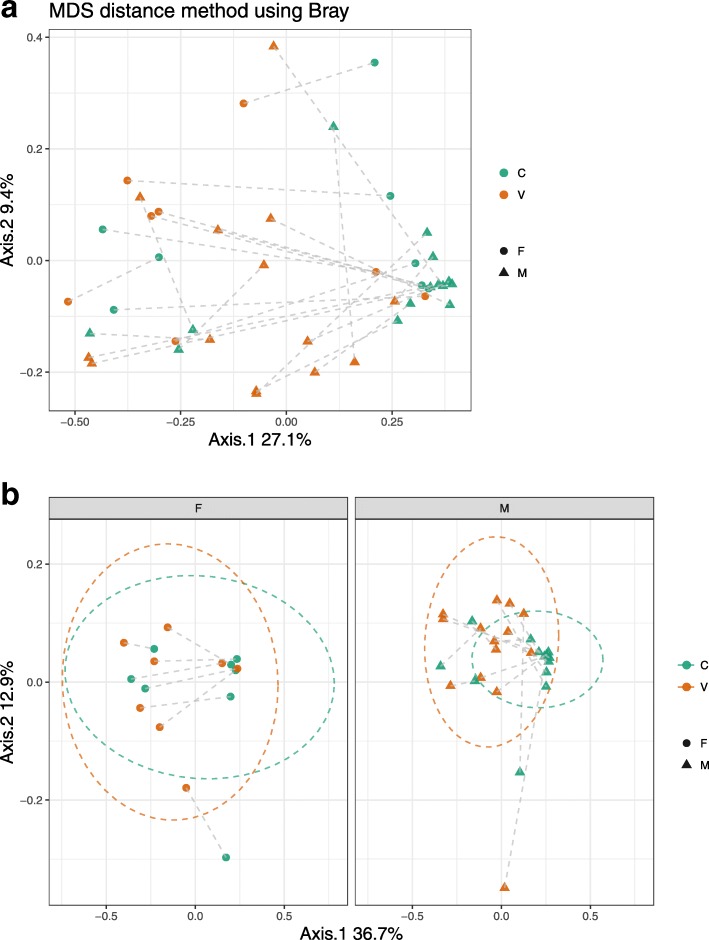
**a** Principal Coordinate Analysis (PCoA) of urine microbiome composition with Bray-Curtis dissimilarity; lines connect urine samples from the same individual. C: cystoscopy; V: voided; F: female; M: male. **b** Principal Coordinate Analysis (PCoA) of urine microbiome composition with Bray-Curtis dissimilarity by sex; lines connect urine samples from the same individual. C: cystoscopy; V: voided; F: female; M: male

When examining beta diversity in all samples (Fig. 2a), there were no significant differences between voided and cystoscopy samples. When split by sex (Fig. 2b), there was a difference in voided and cystoscopy samples from the same individual in males (*p* = 0.006, Adonis test) but not in females (*p* = 0.317, Adonis test). Samples from the same collection method did not cluster together more than samples from the same individual (Additional file 1: Figure S1).

**Fig. 3:**
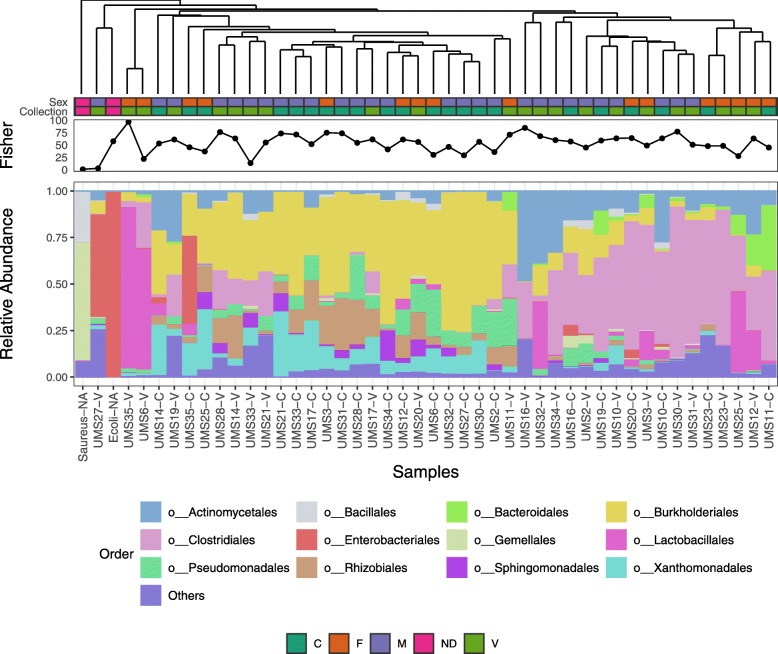
Relative abundance at an order level of samples. In this tree plot, most of the samples can clustered be clustered into two groups: cluster A (shaded in blue; *N* = 22) and cluster B (shaded in brown); *N* = 17). There is no enrichment of the sex or collection method between these two clusters (*p* = 0.11 for Collection, and *p*-value = 0.49 for sex; Fisher’s exact test). Sex of the subject and sample collection approach are indicated by the colored tiles below the tree, where dark green represents for cystoscopy (C), light green for voided (V), orange for female (F), blue for male (M) and pink for not defined (ND, i.e. positive controls)

The relative abundance between taxa in voided and cystoscopy samples was examined. Figure 3 shows the relative abundance at an order level. In this plot, most of the samples can be clustered into two groups and there is no enrichment of the sex or collection method between these two clusters (*p* = 0.11 for Collection and *p* = 0.49 for sex; Fisher’s exact test), suggesting there is no particular pattern associated with sex or the collection approach in the overall relative abundance at the order level.

Even though there is no enrichment detected among the clustering, the plausibility that there is association between collection method or sex and the relative abundance of a particular OTU cannot be excluded. To address this, a mixed-effect regression model was applied for every OTU which has at least 0.1% abundance in at least one sample. Some differences were seen in different taxa between collection method and sex without multiple test correction (Additional file 2: Figure S2, Table 2). However, after multiple corrections with the Benjamini–Hochberg procedure, they did not remain statistically significant.

**Table 2:** Differentially abundant taxa with a raw *p* < 0.05 for the covariates of collection method and sex from the mixed-effect regression model. Note that there is no statistically significance after multiple-testing correction

	Phylum (p)	Class (p)	Order (p)	Family (p)	Genus (p)
Increased in voided samples	Firmicutes (0.012)	Bacilli (0.027)	Lactobacillales (0.037)		
Increased in cystoscopy samples			Xanthomonadales (0.037)	Xanthomonadaceae (0.037)	*Stenotrophomonas* (0.035)
Increased in males	Proteobacteria (0.014)	Betaproteobacteria (0.008	Burkholderiales (0.008)	Comamonadaceae (0.015)	*Tepidimonas* (0.009)
Decreased in males		Bacilli (0.021)	Lactobacillales (0.011)		
				Prevotellaceae (0.006)	*Prevotella* (0.006)
					*Veillonella* (0.004)

## Discussion

This study compared the urinary microbiome from urine samples from the same individual collected by mid-stream voiding and directly from the bladder, by cystoscopy, adding substantively to prior urine microbiome studies. Although there were no differences between collection methods for most microbiome metrics (alpha diversity measures (diversity within a sample) and relative abundance of taxa after multiple correction), beta diversity (diversity between samples) did differ by collection method but for males only.

A possible reason for the difference in beta diversity between collection methods for males only is the longer urethra length in male compared to females. The average length of the male urethra is 20 cm compared with 4 cm in females [21]. Given the shorter length of urethra in females there may be more ascending bacteria entering the bladder, and hence the propensity for females to have urinary tract infections; therefore the urine microbiome of the bladder and urethra may be more similar in females compared with males. Indeed in males with lower urinary tract symptoms, voided specimens contained more lower urinary tract microbes than catheterized specimens [5]. Other sex specific factors to consider include hormonal and sexual activity differences. However, Wolfe et al examined urine samples from adult women only by 16 s rRNA gene sequencing and found that voided urine samples contained mixtures of urinary and genital tract bacteria whereas parallel urine samples collected by transurethral catheter and suprapubic aspirate were similar to each other [17].

Prior to multiple corrections, some differences in the relative abundance of taxa were seen between collection methods and sex. Lactobacillales was both increased in voided samples and in females; a predominance of the order Lactobacillales and genus *Lactobacillus* have been previously reported in the female urine microbiome [22]. However, after multiple corrections there was no statistical significance. Possible differences in taxa abundance between collection methods warrants further exploration in a larger sample size.

Large epidemiological studies are needed to investigate the role of the urinary microbiome in bladder cancer and hence the optimal method for collecting urine for these studies need to be understood. Although there were no statistically significant differences in most microbiome metrics in samples from the same individual collected by mid-stream voided urine and from the bladder by cystoscopy, there were differences in beta diversity in males. This suggests that the urinary microbiome of the two collection methods are not equivalent to each other, at least in males, which is the sex bladder cancer occurs most frequently, and therefore the same collection method should be used within a given study. It is currently unknown whether the urine microbiome from voided urine versus a sample obtained from the bladder would be better as a screening tool for bladder cancer. The urethral microbiome which is likely more reflected in a voided sample, could potentially play a pathogenic role too. For population based studies a voided urine specimen in much easier to collect and has lower associated risks, but the microbiome of this sample is not equivalent to a sample obtained directly from the bladder in males.

Although this is the largest study in bladder cancer of which we are aware that directly compared the urinary microbiome of urine from the samples collected by midstream voiding versus cystoscopy in both sexes, there were several limitations. While the sample size in this study is still relatively small, the ability to compare collection methods in the same individual does increase the strength of this study. In addition, urinary samples generally have a low abundance of bacteria and as with any low abundance microbiomes there is potential for contamination [23]. Our inclusion of both positive and negative controls serves to minimize this concern. While it was documented whether there was antibiotic use within three months of the collected urine sample, using stool microbiome as a reference with substantial recovery within this time period [24], it is unknown if more distant use of antibiotics can have a lasting more pervasive effect on the urine microbiome. Additionally intraurethral lubricant jelly was used on all subjects for cystoscopy; it could be hypothesized that this may transmit distal urethral microbes into the bladder in between voided urine specimen collection and cystoscopic specimen collection.

## Conclusion

There were no differences in urine microbiome metrics of alpha diversity measures and relative abundance of taxa, between specimens collected by midstream voiding versus cystoscopy-. However, there were differences in beta diversity by collection method for males only. This suggests that the urinary microbiomes of the two collection methods are not equivalent to each other, at least in males. These data should be confirmed and within a given study the same collection method should be used.

## Supplementary information

**Additional file 1: Figure S1.** Distances between and within urine samples using different beta diversity metrics.

**Additional file 2: Figure S2.** Circular tree plot of the OTUs with at least 0.1% abundance. Node symbols represent OTUs in different taxonomic rank: kingdom (k), phylum (p), class (c), order (o), family (f), and genus (g). The nodes are colored in pink, blue and green if the OTU has raw *p* < 0.05 for the covariates Collection, Sex, and both, respectively, from the mixed-effect regression model. Note that there is no statistical significance after multiple-testing correction. V: voided; M: male.

## Data Availability

The datasets used and/or analysed during the current study are available from the corresponding author on reasonable request. The 16S rRNA sequence data is deposited in NCBI Sequence Read Archive (SRA); PRJNA600265.

## Abbreviations

OTU: Operational Taxonomic Unit
UC: Urothelial carcinoma

## References

[1] Aragón IM, Herrera-Imbroda B, Queipo-Ortuño MI (2016). The urinary tract microbiome in health and disease. Eur Urol Focus.

[2] Thomas-White K, Kliethermes S, Rickey L (2017). Original research: evaluation of the urinary microbiota of women with uncomplicated stress urinary incontinence. Obstet Gynecol.

[3] Karstens L, Asquith M, Davin S (2016). Does the urinary microbiome play a role in urgency urinary incontinence and its severity?. Front Cell Infect Microbiol.

[4] Pearce MM, Hilt EE, Rosenfeld AB (2014). The female urinary microbiome: a comparison of women with and without urgency urinary incontinence. MBio.

[5] Bajic P, Van Kuiken ME, Burge BK, et al. Male bladder microbiome relates to lower urinary tract symptoms. Eur Urol Focus. 2018;21:S2405-4569(18)30220-7. 10.1016/j.euf.2018.08.001 30143471

[6] Groah SL, Pérez-Losada M, Caldovic L (2016). Redefining healthy urine: a cross-sectional exploratory metagenomic study of people with and without bladder dysfunction. J Urol.

[7] Nelson DE, Van Der Pol B, Dong Q (2010). Characteristic male urine microbiomes associate with asymptomatic sexually transmitted infection. PLoS One.

[8] Siegel R, Ma J, Zou Z (2014). Cancer statistics, 2014. CA Cancer J Clin.

[9] Schwabe RF, Jobin C (2013). The microbiome and cancer. Nat Rev Cancer.

[10] Mostafa MH, Sheweita SA, O'Connor PJ (1999). Relationship between schistosomiasis and bladder cancer. Clin Microbiol Rev.

[11] Alfano M, Canducci F, Nebuloni M (2016). The interplay of extracellular matrix and microbiome in urothelial bladder cancer. Nat Rev Urol.

[12] Xu W, Yang L, Lee P (2014). Mini-review: perspective of the microbiome in the pathogenesis of urothelial carcinoma. Am J Clin Exp Urol.

[13] Redelman-Sidi G, Glickman MS, Bochner BH (2014). The mechanism of action of BCG therapy for bladder cancer--a current perspective. Nat Rev Urol.

[14] Bajic P, Wolfe AJ, Gupta GN (2019). The urinary microbiome: implications in bladder Cancer pathogenesis and therapeutics. Urology..

[15] Bao Y, Razvi H, Gloor G (2016). Mp88-19 urinary microbiome patterns appears to be correlative to intravesical recurrence of non-muscle invasive bladder cancer. J Urol.

[16] Manoni F, Gessoni G, Alessio MG (2011). Mid-stream vs. first-voided urine collection by using automated analyzers for particle examination in healthy subjects: an Italian multicenter study. Clin Chem Lab Med.

[17] Wolfe AJ, Toh E, Shibata N (2012). Evidence of uncultivated bacteria in the adult female bladder. J Clin Microbiol.

[18] Caporaso JG, Kuczynski J, Stombaugh J (2010). QIIME allows analysis of high-throughput community sequencing data. Nat Methods.

[19] McMurdie Paul J., Holmes Susan (2013). phyloseq: An R Package for Reproducible Interactive Analysis and Graphics of Microbiome Census Data. PLoS ONE.

[20] Wong WSW, Clemency N, Klein E (2017). Collection of non-meconium stool on fecal occult blood cards is an effective method for fecal microbiota studies in infants. Microbiome..

[21] Basic human anatomy (2008). O'Rahilly, Müller, Carpenter & Swenson. Chapter 33.

[22] Fouts DE, Pieper R, Szpakowski S (2012). Integrated next-generation sequencing of 16S rDNA and metaproteomics differentiate the healthy urine microbiome from asymptomatic bacteriuria in neuropathic bladder associated with spinal cord injury. J Transl Med.

[23] Bao Y, Al KF, Chanyi RM, Whiteside S (2017). Questions and challenges associated with studying the microbiome of the urinary tract. Ann Transl Med.

[24] Dethlefsen L, Huse S, Sogin ML, Relman DA (2008). The pervasive effects of an antibiotic on the human gut microbiota, as revealed by deep 16S rRNA sequencing. PLoS Biol.

