# Supplementary figures and images for: Studying the urine microbiome in superficial bladder cancer: samples obtained by midstream voiding versus cystoscopy
Source: BMC Urol. 2020 Jan 28;20:5. doi: 10.1186/s12894-020-0576-z (PMC6986141; doi:10.1186/s12894-020-0576-z)

Distance

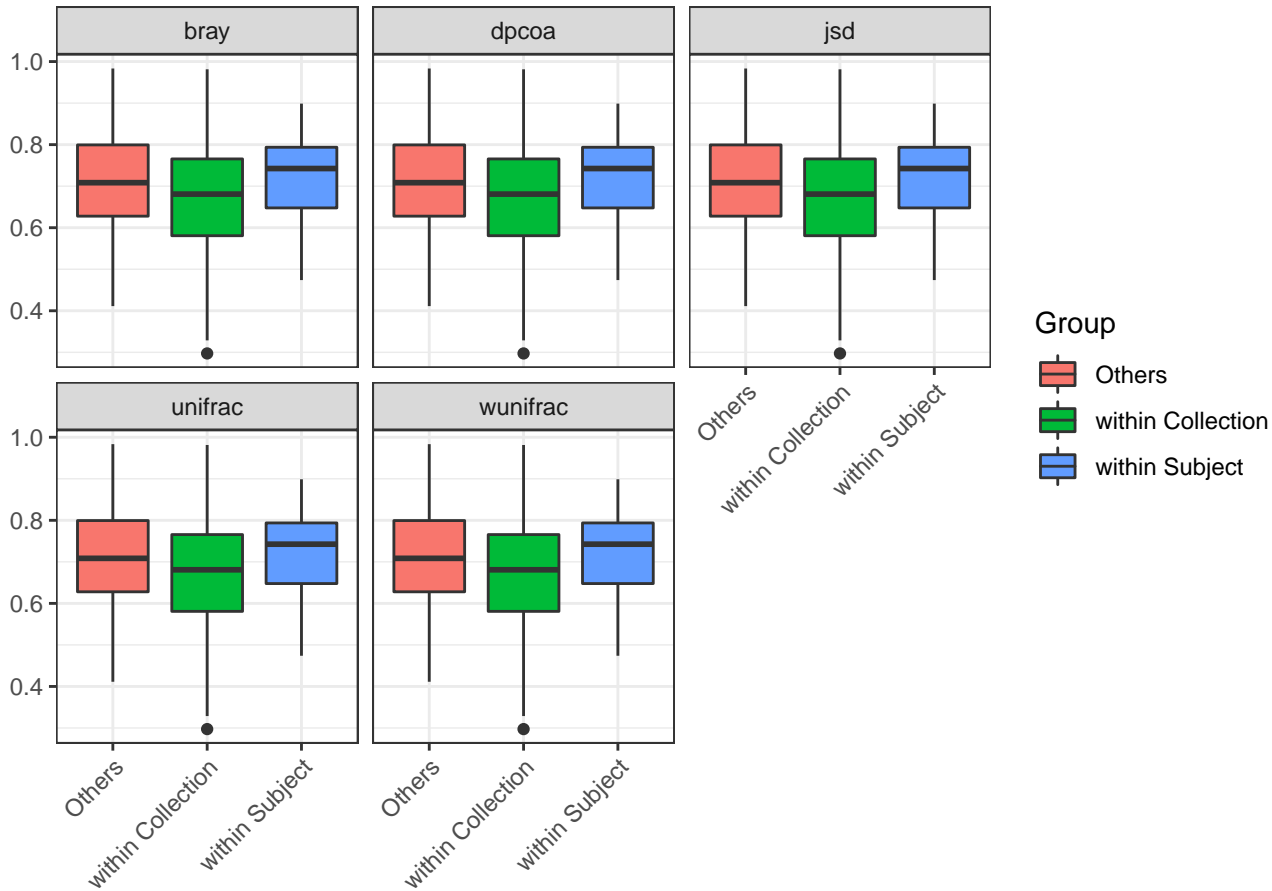

Supplement: Supplementary file 1 — Additional file 1: Figure S1. Distances between and within urine samples using different beta diversity metrics. [file 12894_2020_576_MOESM1_ESM.pdf]

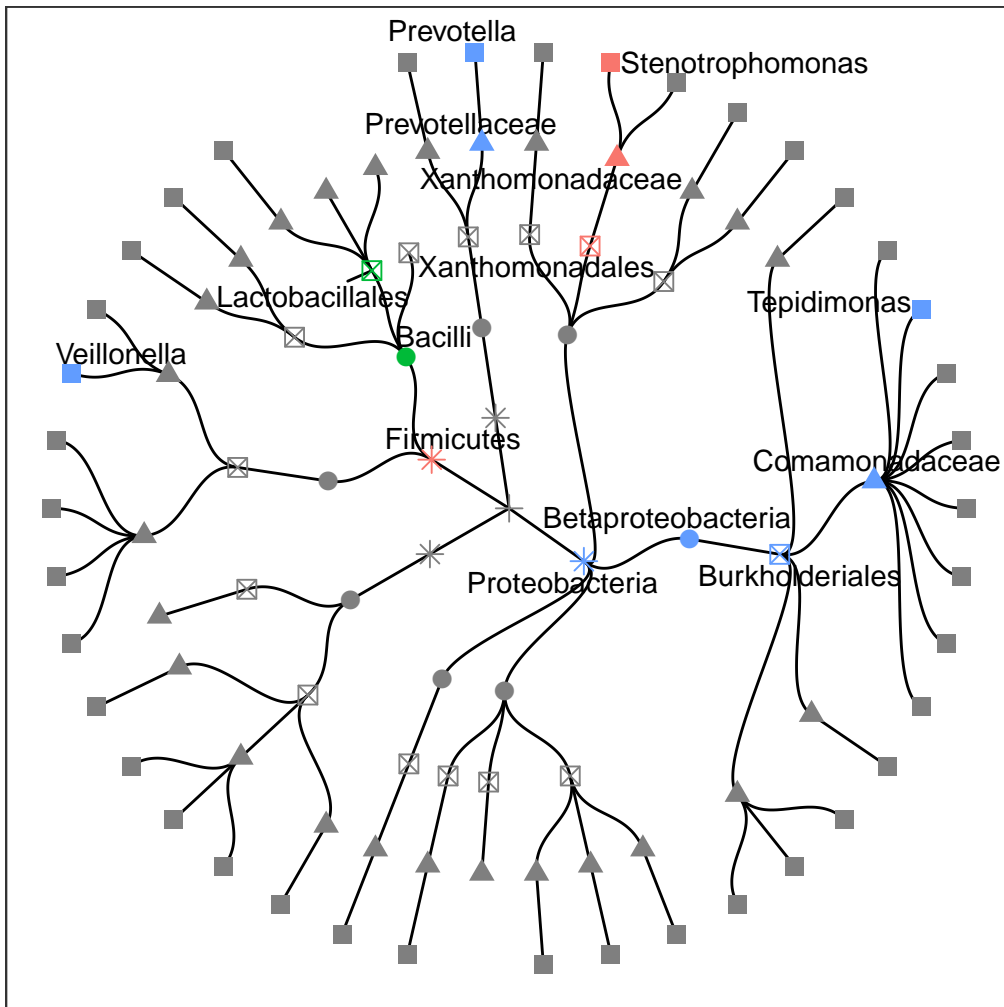

# Term

- CollectionV
- CollectionV/SexM
- SexM
- NA

# Rank

- c
- f
- g
- k
- o
- p

Supplement: Supplementary file 2 — Additional file 2: Figure S2. Circular tree plot of the OTUs with at least 0.1% abundance. Node symbols represent OTUs in different taxonomic rank: kingdom (k), phylum (p), class (c), order (o), family (f), and genus (g). The nodes are colored in pink, blue and green if the OTU has raw p < 0.05 for the covariates Collection, Sex, and both, respectively, from the mixed-effect regression model. Note that there is no statistical significance after multiple-testing correction. V: voided; M: male. [file 12894_2020_576_MOESM2_ESM.pdf]
